# MFD-YOLO: an efficient instance segmentation model for precision monitoring of UAV-based lychee orchards

**DOI:** 10.3389/fpls.2026.1804386

**Published:** 2026-04-10

**Authors:** Sihua Zhi, Jiaqi Zheng, Yangyi Tan, Minjie Hu, Yumeng Feng, Wanjia Yu, Lin Liu

**Affiliations:** 1 School of Information Engineering, Shaoguan University, Shaoguan, China; 2 College of Artificial Intelligence and Low-Altitude Technology, South China Agricultural University, Guangzhou, China; 3 Zhaoqing Federation of Supply and Marketing Cooperative, Zhaoqing, China; 4 College of Life Sciences, South China Agricultural University, Guangzhou, China; 5 School of Computing Science, University of Glasgow, Glasgow, Scotland, United Kingdom

**Keywords:** computer vision, efficient convolutional networks, efficient neural networks, instance segmentation, precision agriculture, UAV-borne image processing

## Abstract

Accurate instance segmentation of lychee canopy regions is fundamental for precision orchard management. UAV-based monitoring faces a critical dilemma: the heavy computation required for complex canopy features conflicts with the limited resources of edge devices. To resolve this accuracy-efficiency trade-off, we propose MFD-YOLO (Multi-scale-Downsampling Decoupling), a model designed for real-time UAV monitoring in orchard environments that addresses the limitations of conventional models, namely single-scale feature representation and insufficient inference efficiency. The main contributions are as follows: (1) Addressing the challenge of indistinct canopy boundaries and complex textures, we design the Multi-scale Feature Extraction (MFE) block. By employing a multi-branch parallel structure during training to capture both global contours and fine-grained leaf details, and re-parameterizing them into a single layer for inference, we enhance feature representation without incurring extra latency. (2) Addressing the issue where fine edge details are lost during standard downsampling, we introduce the Spatial-Channel Decoupled (SD) module. Unlike traditional strided convolutions that compress dimensions simultaneously, SD prioritizes channel information adaptation before spatial reduction, effectively preserving small-object features while reducing redundancy. (3) Evaluated by mAP50, precision, and recall metrics, the MFD-YOLO model performs excellently in lychee canopy segmentation tasks. It simultaneously outputs bounding box coordinates for real-time coarse localization and pixel-level masks for precise canopy delineation in complex orchard scenarios, effectively addressing practical issues in complex orchard field environments while achieving low latency on the server side. The dataset is randomly partitioned into training and validation sets at a ratio of 7:3. This model provides reliable technical support for key links including scientific pesticide application, targeted pruning, and efficient harvesting.

## 1 Introduction

**Figure 1:**
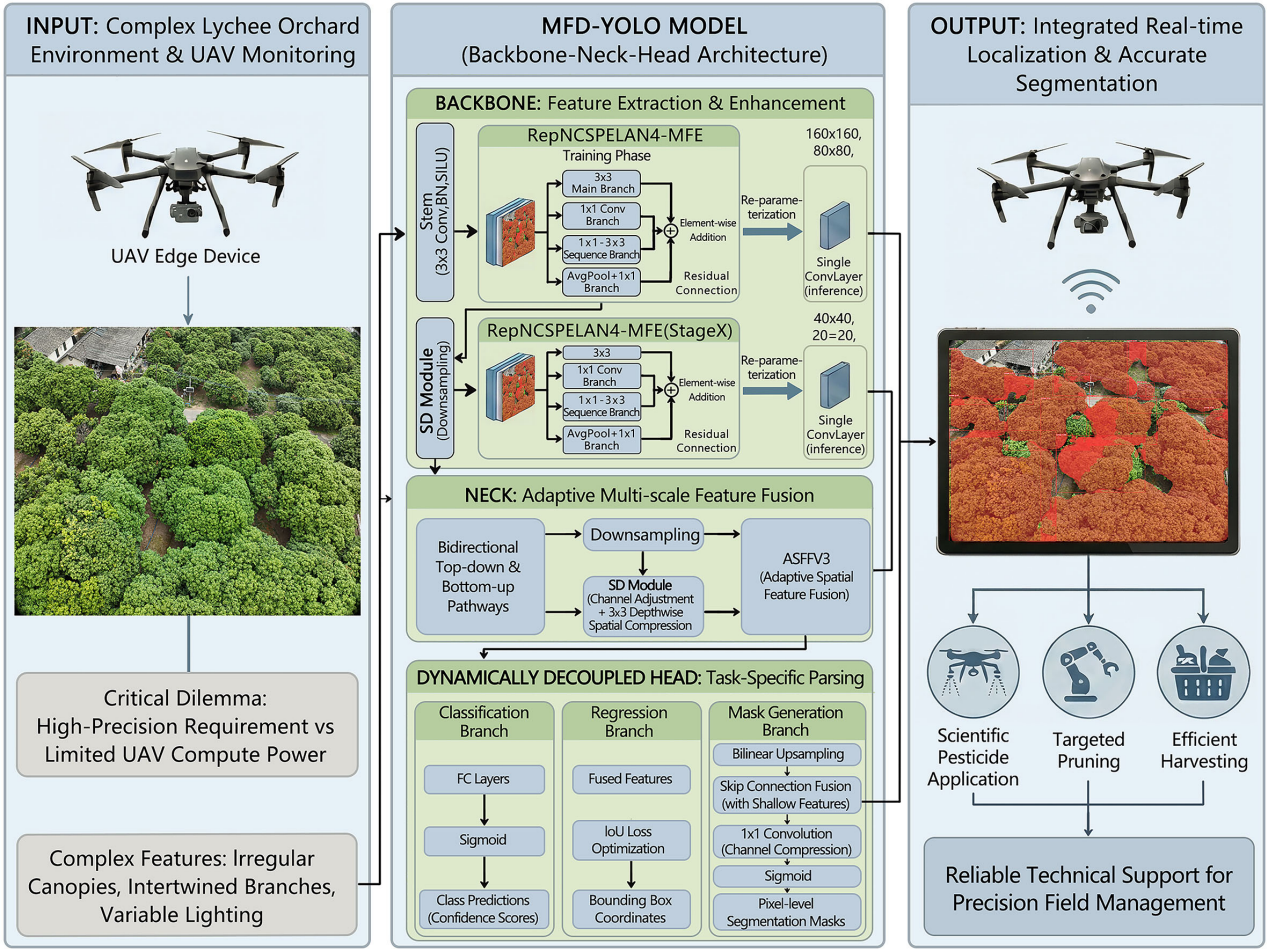
Technical roadmap and workflow. In lychee orchards in Guangzhou, Guangdong Province, a DJI M300 RTK collected multi-angle RGB images to capture complex canopy details. The proposed MFD-YOLO model leverages a re-parameterized backbone and decoupled heads to accurately segment fruit trees within the field of view. This establishes a robust visual algorithm foundation for mapping geodetic coordinates, further serving critical application scenarios such as waypoint planning and scientific pesticide application.

Lychee is the leading fruit crop in China’s tropical and subtropical regions, with a long history of commercial cultivation and extensive planting areas. Owing to its distinctive flavor, lychee is favored by consumers worldwide. However, lychee exhibits relatively weak disease resistance, which leads to pronounced year-to-year yield fluctuations (Qi et al., 2023). With the rapid development of precision agriculture, the deep integration of unmanned aerial vehicles (UAVs) and agricultural practice has emerged. Thanks to their high flexibility, operational precision, and intelligent capabilities, UAVs show great potential in precision agriculture. In this context, building a model that can accurately evaluate lychee canopy structure is particularly important for enabling precision pesticide application, intelligent pest and disease detection, and efficient post-harvest management in lychee orchards. The technical approach is outlined in **Figure 1**.

Against the background of deep UAV–agriculture integration, deep learning has become a core technical enabler in agricultural remote sensing because of its strengths in extracting complex image features and modeling unstructured information. For example, Wang Xuewen et al. combined airborne multispectral UAV imagery with the DeepLabv3+ semantic segmentation model (Wang et al., 2020), using the Optimal Index Factor (OIF) to select the best three-band combination as model input and thereby improving segmentation accuracy; however, their work did not systematically address spectral redundancy in multispectral images, and raw multi-band data can increase computational burden. Lin Zhaowei proposed SANet, a multimodal semantic segmentation model based on spatial–spectral attention (Lin, 2023), which adaptively fuses UAV multispectral and RGB images to achieve high-precision segmentation of field boundaries and crop types, but its feature adaptation to complex terrain remains limited. For the intelligent management of lychee orchards, the MDP-Model (Lu et al., 2024), based on the Bayesian decision fusion algorithm with adaptive weights, fuses multimodal data to overcome the limitations of a single data source. However, these multimodal approaches often require complex sensor payloads, increasing the cost and weight of UAVs.

The main contributions of this work are summarised as follows: (1) We designed the Multiscale Feature Extraction (MFE) block to resolve the trade-off between capturing irregular canopy details and maintaining inference speed. By employing a parallel multi-branch structure during training, the block captures features ranging from global contours to fine-grained leaf textures. These branches are mathematically merged into a single convolutional layer for inference via re-parameterization, enhancing feature representation without incurring extra latency. (2) We developed the Spatial-Channel Decoupled (SD) module to prevent the loss of fine edge details that typically occurs during standard downsampling. Unlike traditional strided convolutions that compress spatial and channel dimensions simultaneously, this module prioritises channel adaptation before spatial reduction. This decoupled strategy effectively preserves faint features of small targets while minimising computational redundancy for efficient deployment. (3) The MFD-YOLO model supports precision orchard management tasks. It achieves an integrated output of high-precision segmentation and real-time localization, even in challenging environments. These results provide reliable and actionable data for downstream agricultural operations, such as scientific pesticide application, targeted pruning, and yield estimation.

This Introduction has framed the precision-management needs of lychee orchards and the current state of related technologies, identified gaps in existing research, and motivated the MFD-YOLO design under orchard complexity and UAV edge-deployment constraints. Chapter 2 details the methodological foundations and methods, including the dataset characteristics, the MFD-YOLO network architecture, and the designs of the MFE block and SD module. Chapter 3 presents experimental results and evaluates model performance and practical feasibility. Chapter 4 discusses practical application value and proposes future directions such as expanding multi-species datasets and applying few-shot transfer learning. Chapter 5 concludes the study and outlines prospects for future research.

## 2 Related work

### 2.1 General semantic segmentation models

Classical CNN models have laid the technical foundations for agricultural visual recognition, producing key advances in feature extraction, scale recovery, and training of deep networks. FCN (Long et al., 2015) pioneered semantic segmentation by converting the fully connected layers at the back end of conventional CNNs into convolutions and using skip connections to fuse multi-level features. SegNet (Badrinarayanan et al., 2017) stores the indices from max-pooling and uses them in unpooling to enable learned recovery of pooled information during upsampling, mitigating the scale-reconstruction blurring associated with naïve deconvolution. ResNet (He et al., 2016) and its variants (Xie et al., 2016), through residual connections, overcame the gradient-vanishing bottleneck in deep networks and allowed effective use of semantics accumulated through downsampling. Nevertheless, these models rely on local receptive-field mechanisms defined by single convolutional kernels, which limits the dimensionality of feature expression and makes it difficult for a fixed receptive field to cover the large scale variations commonly present in agricultural scenes.

As segmentation techniques have evolved, their applications to complex agricultural tasks have expanded. Mask R-CNN (He et al., 2017) augments Faster R-CNN (Ren et al., 2017) with a mask branch to enable instance-level segmentation in addition to bounding-box detection; U-Net (Ronneberger et al., 2015) and its variants (Zhou et al., 2018) enhance the fusion of shallow texture features and deep semantic features through a symmetric encoder-decoder structure and dense skip connections; PSPNet (Zhao et al., 2016) introduces a pyramid pooling module to aggregate and fuse contextual information at multiple scales, compensating for CNNs’ limited global perception. However, these models are difficult to deploy for real-time UAV monitoring in orchards and show weaknesses in capturing fine-grained structures such as canopy edges.

### 2.2 Efficient networks and re-parameterization

To address constrained feature extraction and low inference efficiency, researchers have proposed various improvements. ACNet (Ji et al., 2020) decouples training and inference to optimize feature extraction and deployment efficiency, avoiding extra inference cost. However, the added training-stage complexity can increase the risk of overfitting when annotated data are limited or scenes are homogeneous. ShuffleNet (Zhang et al., 2017) introduces a channel shuffle mechanism to alleviate the inter-channel information isolation caused by group convolution, thus improving the feature representation efficiency of models. RepVGG (Ding et al., 2021) adopts a multi-branch re-parameterization paradigm that substantially expands the feature space of a single convolution without increasing inference parameters or FLOPs; yet diverse branches can amplify local noise and weaken effective feature signals. The MobileNet family (Howard et al., 2017) (Sandler et al., 2018) (Howard et al., 2020) achieves a lightweight design through depthwise separable convolutions —decomposing standard convolutions into depthwise and pointwise operations—which greatly reduces parameters and computation while retaining basic feature extraction capability; however, its cross-channel information exchange is relatively weak, limiting fine-grained feature capture and adaptability.

In UAV-based visual recognition, the central challenge lies in balancing hardware constraints, environmental interference, and recognition accuracy. Constrained by onboard computing power and flight endurance, UAVs cannot support models with massive parameters and complex architectures, yet they must satisfy the real-time requirements of orchard inspection. Consequently, traditional models often fail to achieve an optimal trade-off between efficient design and high accuracy. Furthermore, environmental variability exacerbates this tension: fluctuating flight attitudes prevent a fixed viewpoint relative to the canopy, while temporal variations in illumination induce brightness and color shifts. Moreover, lychee canopies vary greatly in size, present heavy branch intertwining, and exhibit significant density differences across growth stages. These factors demand robust capabilities in multi-scale feature adaptation, occlusion resilience, and edge extraction.

### 2.3 Recent agricultural vision detection and segmentation models

In recent years, deep learning-based object detection and segmentation models have made significant progress in visual monitoring tasks within agricultural scenarios. To address the challenges posed by complex agricultural environments, researchers have conducted in-depth, customized explorations of the YOLO series and the novel Mamba architecture. In the field of agricultural object detection, recent studies have focused on model optimization tailored to the physiological characteristics of specific crops. For example, ROSE-YOLO Zhao et al. (2025) and Succulent-YOLO Li et al. (2025) target the unique morphological features of roses and succulent plants, respectively, effectively improving detection accuracy in complex occluded environments by introducing efficient attention mechanisms and multi-scale feature fusion. The Mamba architecture has also begun to emerge in agricultural vision tasks, leveraging its advantage of capturing long-range dependencies with linear complexity. For instance, Rose-Mamba-YOLO You et al. (2025) combines the State Space Model (SSM) with the YOLO architecture, significantly enhancing the model’s ability to perceive global contextual information while controlling computational resource consumption. In agricultural segmentation tasks based on UAV imagery, several studies have also achieved new breakthroughs. Tao et al. Tao et al. (2025) proposed an innovative precision agriculture framework that combines UAV remote sensing technology with deep neural networks to overcome irregular plant boundaries, realizing high-precision segmentation of weeds and crops. Furthermore, Zhao et al. Zhao et al. (2025) utilized a Mamba-based state space architecture to achieve efficient pixel-level monitoring and segmentation of blueberry maturity, demonstrating the superiority of a global receptive field in extracting fine-grained agricultural features.

Although these models exhibit excellent performance in accuracy and feature extraction capabilities, they still face severe challenges when confronted with UAV edge deployment. In the pursuit of extreme accuracy, the introduction of complex modules in some models easily leads to a surge in parameter count. On edge devices with constrained computational power, the high Memory Access Cost (MAC) associated with a massive underlying architecture can easily lead to a decrease in inference speed, severely compromising model efficiency. To address the aforementioned issues, this paper proposes MFD-YOLO, a deployment-friendly and efficient improved model.

## 3 Materials and methods

### 3.1 Materials of dataset

With continual improvements in UAV performance, the use of unmanned aerial systems in agricultural production has steadily increased; agricultural UAVs have become an indispensable component of smart agriculture. Although mainstream segmentation models such as the DeepLab family (Chen et al., 2016, Chen et al., 2017) achieve high segmentation accuracy, they commonly suffer from lost boundary details, limited robustness in complex scenes, and large model sizes. To meet the deployment requirements of MFD-YOLO in real agricultural scenarios, we constructed a dedicated dataset of UAV-acquired RGB images of lychee trees. The dataset contains 998 images captured at a resolution of 5280 × 3956 pixels, comprising 700 images for the training set and 298 for the validation set. The surveyed orchard is located in Conghua District, Guangzhou City, Guangdong Province, and the image acquisition was conducted in November 2023. Images were collected across multiple growth stages and planting densities, and they reflect orchard terrain variation and diverse canopy morphologies, providing rich scene diversity for model training.

**Figure 2:**
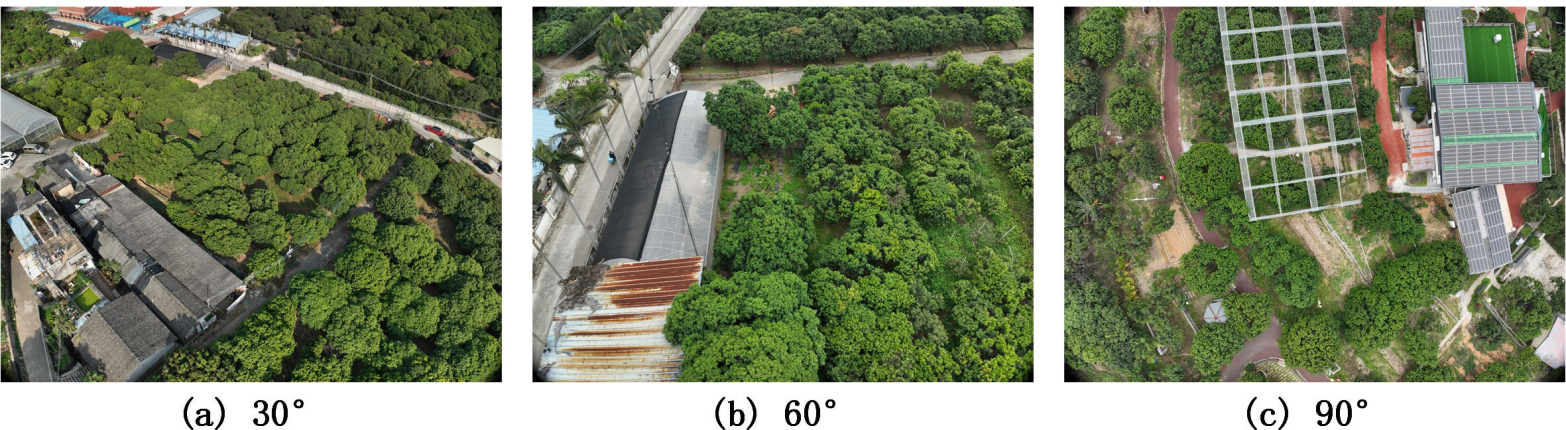
**(a)** 30° downward-looking UAV images; **(b)** 60° downward-looking UAV images; **(c)** 90° downward-looking UAV images.

During the data acquisition process, we utilized a DJI M300 RTK drone equipped with a Zenmuse P1 full-frame photogrammetry camera (45 megapixels, 4.4 m pixel size, 24 mm prime lens). Camera calibration utilized the factory intrinsic parameters, which were further optimized during the aerial triangulation stage. Real-Time Kinematic (RTK) differential positioning was enabled during flight, synchronously recording the camera’s precise position (horizontal accuracy of ±1 cm + 1 ppm) and gimbal attitude at the exact moment of exposure, writing these directly into the image metadata. Subsequently, the images and RTK data were imported into DJI Terra software for feature matching and bundle block adjustment, accurately calculating the exterior orientation parameters for each image. Finally, based on the aerial triangulation results, a Digital Orthophoto Map (DOM) was generated within the CGCS2000 Gauss-Kruger projection coordinate system, seamlessly establishing the correspondence between pixel coordinates and geographic coordinates. Furthermore, we employed a targeted multi-angle photographing strategy (approximately 30°, 60° and 90°) **Figure 2** to ensure complete capture of canopy tops, sides and locally occluded regions. By establishing this precise mapping relationship between pixel coordinates and the geographic coordinate system, the dataset can synchronously output exact geolocation information for lychee canopies. This georeferenced annotation provides a high-quality labeling foundation for the segmentation task of the MFD-YOLO model, and supports downstream tasks that require spatially explicit outputs.

### 3.2 MFD-YOLO network

**Figure 3:**
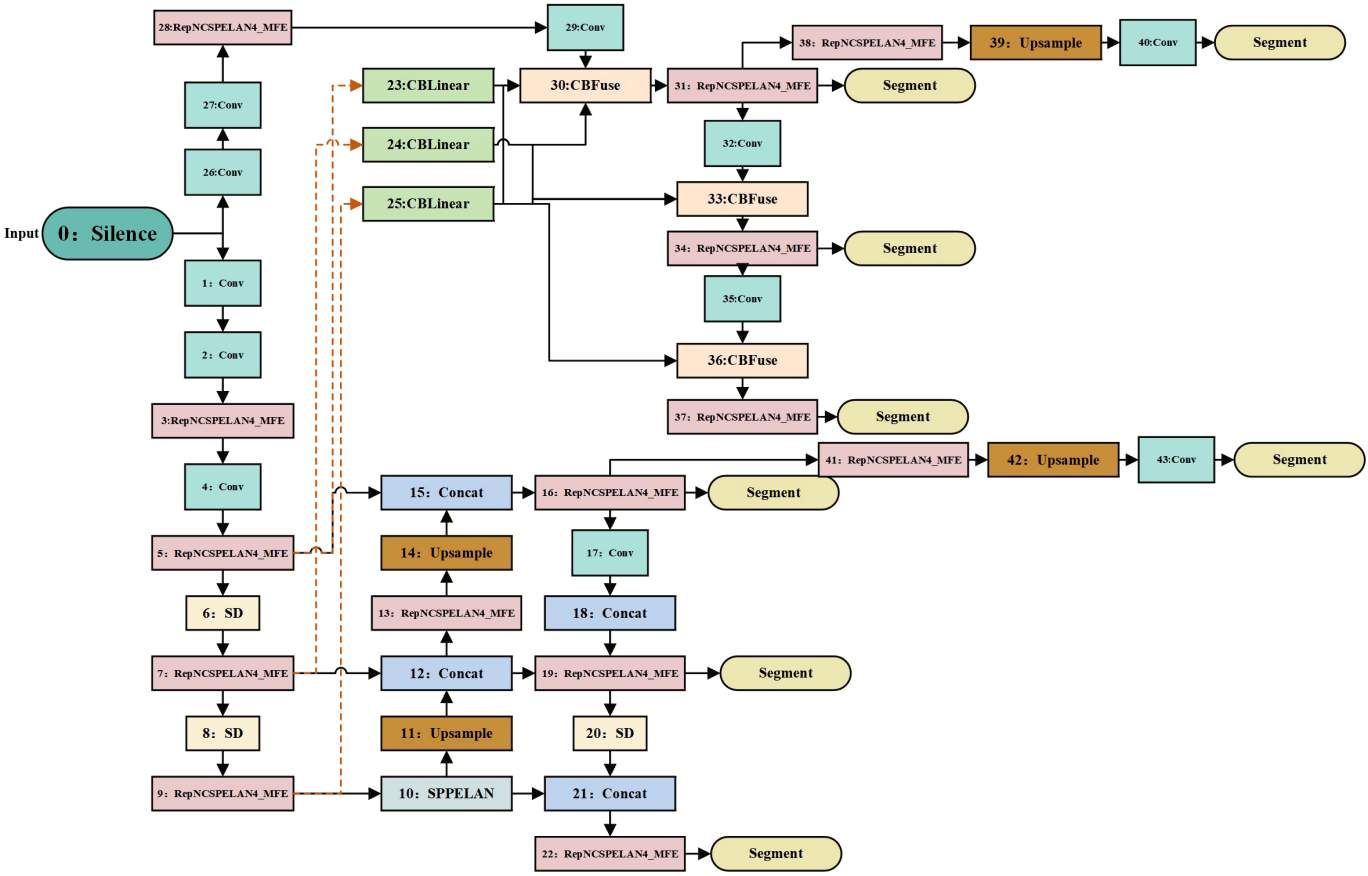
The architecture of MFD-YOLO. The network consists of three main parts: Backbone, Neck, and Head. All improved components and processes are clearly marked in the figure.

To adapt to UAV-borne lychee canopy recognition, we propose an efficient network, MFDYOLO (**Figure 3**), whose architecture is composed of three parts: a backbone, a neck, and a head. The backbone is responsible for feature extraction and enhancement; the neck focuses on adaptive multi-scale feature fusion to improve representation of small and occluded targets; and the head parses the enhanced features from the backbone and neck to output class predictions, bounding boxes and pixel-level segmentation masks. To address the common trade-off where high accuracy is achieved at the cost of model heaviness—making deployment on UAV edge hardware difficult—MFDYOLO balances accuracy and efficiency via two core modules. In the backbone, the conventional convolutional layers inside YOLOv9’s RepNCSPELAN block are replaced by the MFE block, which uses a multi-branch structure during training to enrich feature diversity and is equivalently transformed into a single convolutional layer at inference via six types of re-parameterization transformations. During downsampling in the backbone and in coordination with the MFE block, an SD (Spatial–Channel Decoupled) module is introduced in the neck’s ASFFv3 cross-scale fusion stage. The SD module uses a decoupled design of “1×1 pointwise channel adjustment + 3×3 depthwise downsampling”, which reduces small-object and occlusion-related feature loss while directly lowering computation and parameter count, thereby preserving the model’s efficient nature for UAV deployment.

Given preset hyperparameters, three-channel RGB images of lychee trees are first processed by the backbone stem, where a 3×3 convolution performs initial feature extraction and dimensionality reduction. This is immediately followed by batch normalization to eliminate distribution shifts and a SiLU activation function to inject nonlinear responses. In the standard YOLOv9 architecture, the foundational building block is the RepNCSPELAN4 module, which relies on conventional convolutional layers. While efficient, this standard design struggles to simultaneously capture the global contours of lychee trees and the fine-grained textures of foliage and branches. Addressing this limitation, we re-engineered this core module by embedding our proposed MFE Blocks to replace the original internal convolutional units; this novel structure is designated as RepNCSPELAN4-MFE. Within each backbone stage, the RepNCSPELAN4-MFE module processes features via a four-branch parallel structure: a 3×3 main branch captures mid-scale structural foundations; a 1×1 convolution branch focuses on local fine-grained details; a sequential 1×1 + 3×3 branch expands the receptive field to capture correlated features in occluded regions; and an average-pooling plus 1×1 convolution branch extracts global context. The outputs from these branches are fused via element-wise addition and then mathematically converted into a single convolutional layer using re-parameterization techniques. Finally, a residual connection adds the transformed output back to the original input, generating enhanced feature maps that combine multi-dimensional expressiveness with gradient continuity. Concurrently, during the backbone’s scale compression phase, SD Modules are employed to reduce feature resolution, synchronously outputting multi-scale feature representations ranging from 160×160 down to 20×20.

The neck component prioritizes fusing features from diverse scales to bolster the detection of small and occluded targets. Multi-scale features from the backbone first traverse a neck module containing bidirectional bottom-up and top-down paths to achieve preliminary cross-scale alignment. For feature downsampling, we extend the efficiency optimization of the backbone by employing the SD Module. This module first executes channel dimension adjustment and redundancy removal, followed by spatial resolution compression via a 3×3 depthwise convolution (where the number of groups equals the output channels). The strategic insertion of SD Modules at specific stages in both the backbone and neck ensures consistency in efficient downsampling and feature retention throughout the architecture. This design not only reduces computational load through the efficient nature of depthwise convolution but also maximizes the retention of faint edge information—such as lychee fruits and branches—that would otherwise be lost. Subsequently, the ASFFv3 module applies dynamic weighting to the processed multi-scale features, learning spatially adaptive weights to balance the contributions of shallow details and deep semantics. The result is an aggregated feature set characterized by both detailed integrity and semantic consistency, providing high-quality input for the head.

Finally, the three key scale features output by the neck are fed into a dynamic decoupled head, where three task branches operate synchronously. The classification branch extracts deep semantic features mapped through fully connected layers with sigmoid activation to output canopy class confidence, distinguishing targets from the background. The regression branch predicts canopy bounding box coordinates based on fused multi-scale features, employing an IoU loss to iteratively optimize localization error and provide coarse localization references for segmentation. The mask generation branch focuses on pixel-level segmentation: it performs lightweight bilinear upsampling on input features, fuses them with shallow detail features via skip connections, compresses channel redundancy through a 1×1 convolution, and finally produces binary segmentation masks via an element-wise sigmoid activation to precisely delineate canopy pixel regions. The network simultaneously outputs class predictions, bounding box coordinates, and pixel-level masks. This integrated output capability allows the model to not only locate lychee trees in real-time but also precisely depict their canopy coverage, providing essential data for downstream tasks. The training pseudocode for MFD-YOLO is shown in **Algorithm 1**.

Algorithm 1

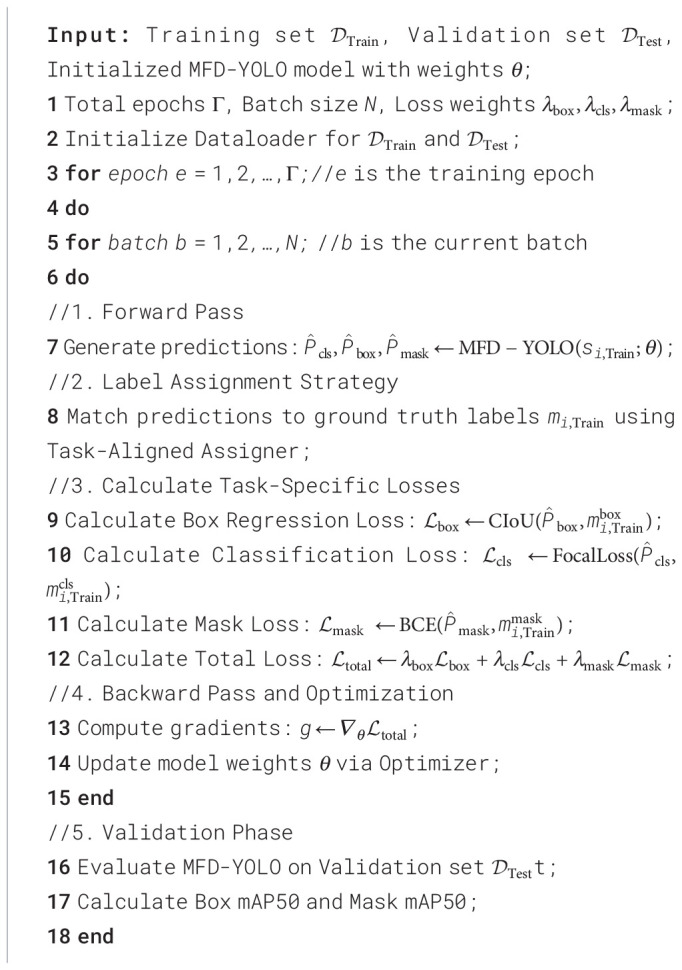

### 3.3 MFE block

To ensure that UAVs can achieve high accuracy in practical visual tasks while also improving inference efficiency, researchers have explored various approaches. The Inception family (Szegedy et al., 2014) (Ioffe and Szegedy, 2015) (Szegedy et al., 2016) (Szegedy et al., 2016a) (Chollet, 2017) uses multiple convolutional kernels of different sizes and pooling operations within the same network layer; by fusing multi-scale branches it enhances feature diversity and represents one of the earlier multi-branch architectures, but its inference structure is complex and slow, making it difficult to adapt to practical tasks.

In complex lychee orchard environments, the canopy morphology varies drastically due to uneven growth and intertwined branches. Capturing these features requires a network with a multi-scale receptive field: large kernels are needed for the overall tree shape, while small kernels are essential for leaf edges and gaps. However, simply stacking multi-scale branches creates a heavy computational burden unsuitable for real-time UAV deployment. And Standard convolutional layers rely on fixed receptive fields, which limits their ability to simultaneously capture global canopy contours and fine-grained leaf textures.

**Figure 4:**
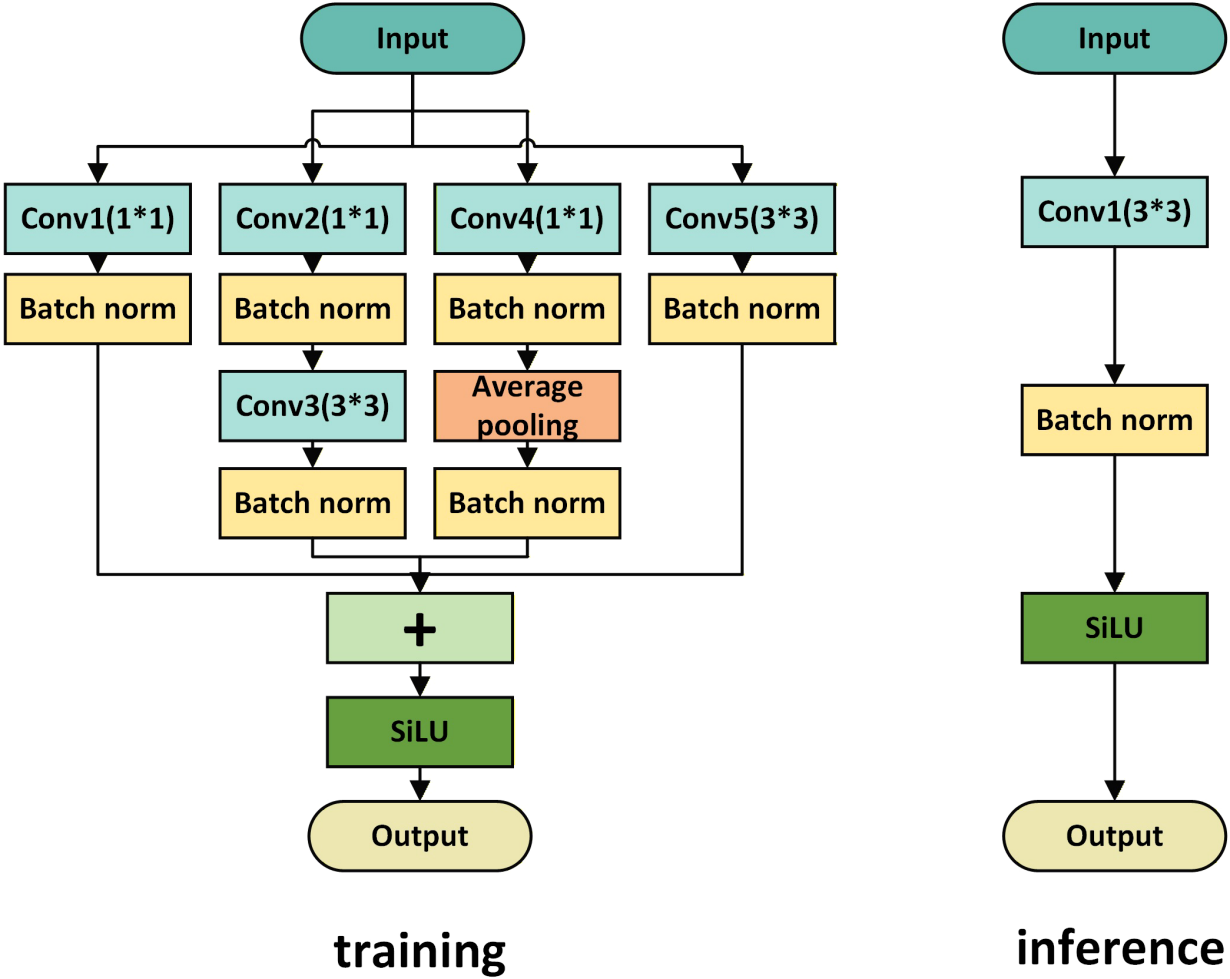
Two structures of the MFE block. The multi-branch structure of MFE block during training and its merging into a single convolution layer during inference.

**Figure 5:**
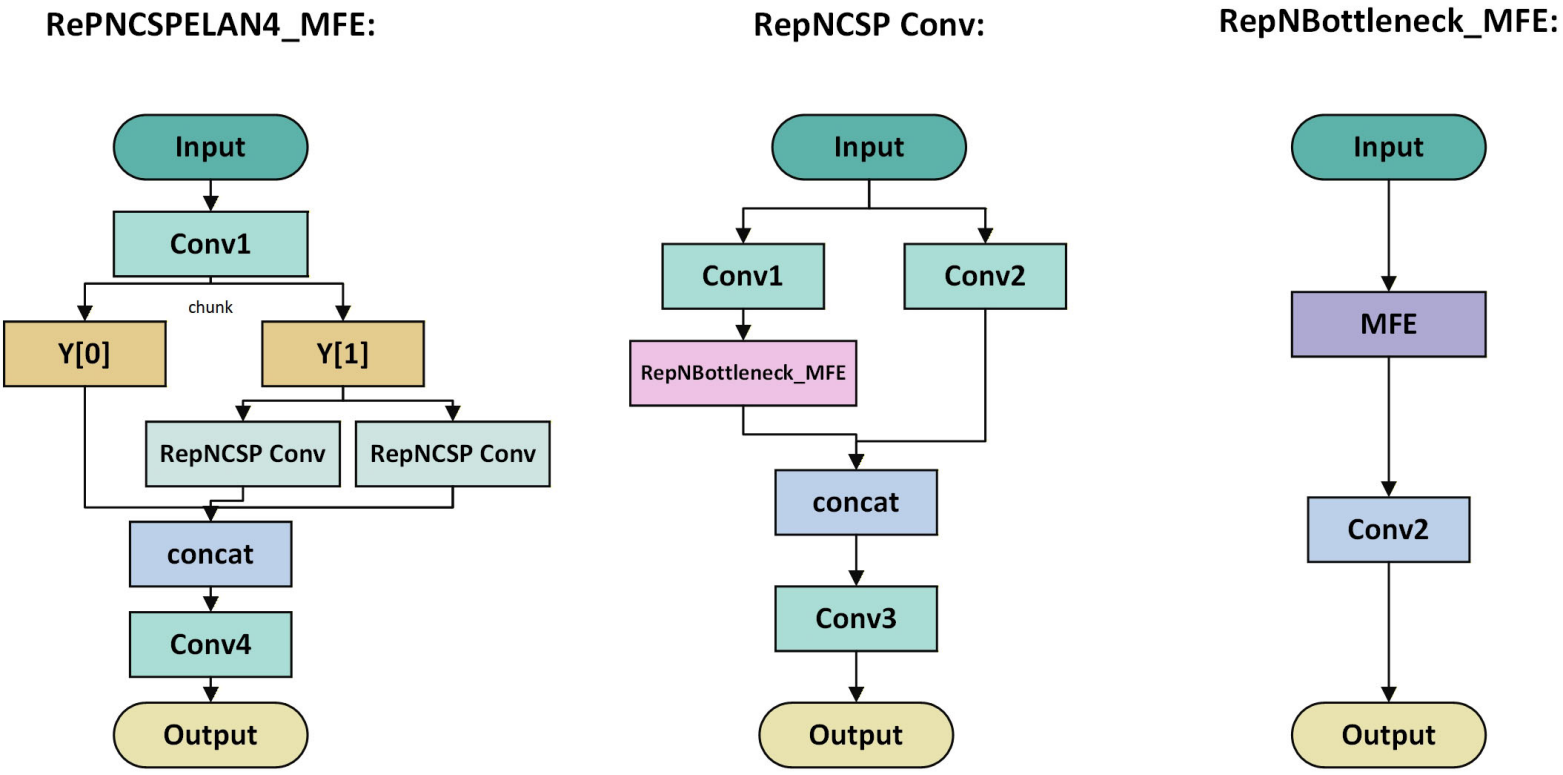
The structure of the RePNCSPELAN4-MFE module is shown in the figure.

To balance ‘fine-grained feature capture’ during training and ‘high-speed inference’ during deployment, we propose the MFE block (**Figure 4**) based on structural re-parameterization. Although existing re-parameterization techniques have been extensively explored in models such as RepVGG and ACNet, these architectures are primarily tailored for general image classification tasks. They typically rely on standard 3 × 3, 1 × 1, and identity mapping branches, which lack the adequate receptive field required to capture global density information and handle deep occlusions. Consequently, applying them directly to the complex structures of lychee canopies yields suboptimal results. In this study, the MFE block is composed of convolutional layers of different sizes and an average-pooling branch: these layers operate in parallel to capture different feature representations and are ultimately merged. After training, these complex structures are combined and simplified into a single convolutional layer through six methods so as not to introduce additional computational burden during inference. Rather than merely appending the re-parameterization module to the end of the backbone, the specific implementation of the MFE block in the model relies on combining with the RePNCSPELAN4 module to form the RePNCSPELAN4-MFE module, whose structure is illustrated in **Figure 5**.

To achieve a substantial improvement in feature diversity via the cooperative effects of differentiated paths, the MFE block is designed with a four-branch parallel feature-extraction structure. The main branch is a K×K convolution branch, implemented as a 3×3 convolution followed by a batch normalization (BN) layer as the basic feature-extraction unit; it captures mid-scale structural features of the tree canopy and can be mathematically expressed as Equation 1.

(1) $$F_{\text{base}}(X)=\text{BN}(W_{3\times 3}*X+b_{3\times 3})1$$

Among them, $W_{3\times 3}\in {\mathbb{R}}^{C_{\text{out}}\times C_{\text{in}}\times 3\times 3}$ denotes the convolution kernel, ∗ represents the standard discrete convolution operator, and BN (·) Ioffe and Szegedy (2015) denotes the batch normalization (BN) operation.

The second branch is a 1×1 convolutional branch: a 1×1 convolution followed by a batch normalization (BN) layer focuses on local fine-grained features, such as lychee leaf-edge textures and small inter-leaf gaps. Its feature response can be mathematically expressed as Equation 2.

(2) $$F_{1\times 1}(X)=\text{BN}(W_{1\times 1}*X+b_{1\times 1})$$

This branch compresses the channel dimension *W*_1_×_1_ ∈ R*^C^*^out^×*^C^*^in^×^1^×^1^ to improve computational efficiency while enhancing nonlinear interactions across channels.

The third branch is the average-pooling branch, consisting of a ‘K×K average-pooling + 1×1 convolution + BN’ sequence. By aggregating global contextual information, this branch suppresses local noise and captures global features such as canopy density distribution. Its feature response can be expressed mathematically as Equation 3.

(3) $$F_{\text{pool}}(X)=\text{BN}(W_{1\times 1}^{\text{pool}}*(\text{AvgPool}(X))+b_{\text{pool}})$$

Among them, AvgPool (·) denotes the average-pooling operation, which is equivalent to a special convolution with weights 1/*K*^2^.

The fourth branch is a 1×1–K×K sequential branch, in which a 1×1 convolution is concatenated with a 3×3 convolution to expand the receptive field and capture correlation features in occluded regions. Its feature mapping can be expressed mathematically as Equation 4.

(4) $$F_{\text{seq}}(X)={\text{BN}}_{2}(W_{3\times 3}^{\text{seq}}*({\text{BN}}_{1}(W_{1\times 1}^{\text{seq}}*X+b_{\text{seq}1}))+b_{\text{seq}2})$$

This branch dynamically adjusts the intermediate-channel quantity $C_{\text{mid}}=\alpha \cdot C_{\text{in}}$ (*α* denotes the expansion coefficient) to balance receptive-field enlargement and computational cost.

The outputs of the branches are fused by element-wise addition to form a multi-dimensional feature aggregation Equation 5.

(5) $$F_{\text{MFE}}(X)=\sigma (F_{\text{base}}(X)+F_{1\times 1}(X)+F_{\text{pool}}(X)+F_{\text{seq}}(X))$$

Here, *σ* (·) denotes the SiLU Elfwing et al. (2018) activation function, which injects nonlinearity to enhance the model’s capacity to fit complex features. Compared with conventional single-branch convolution, this multi-branch design offers a fully covered receptive field that addresses the inability of a single convolution to capture features of multi-scale targets; additionally, its diversified feature dimension outputs can simultaneously encode a variety of feature information. So as to ensure that the edge features of lychee canopy can be accurately divided in different scenarios.

**Figure 6:**
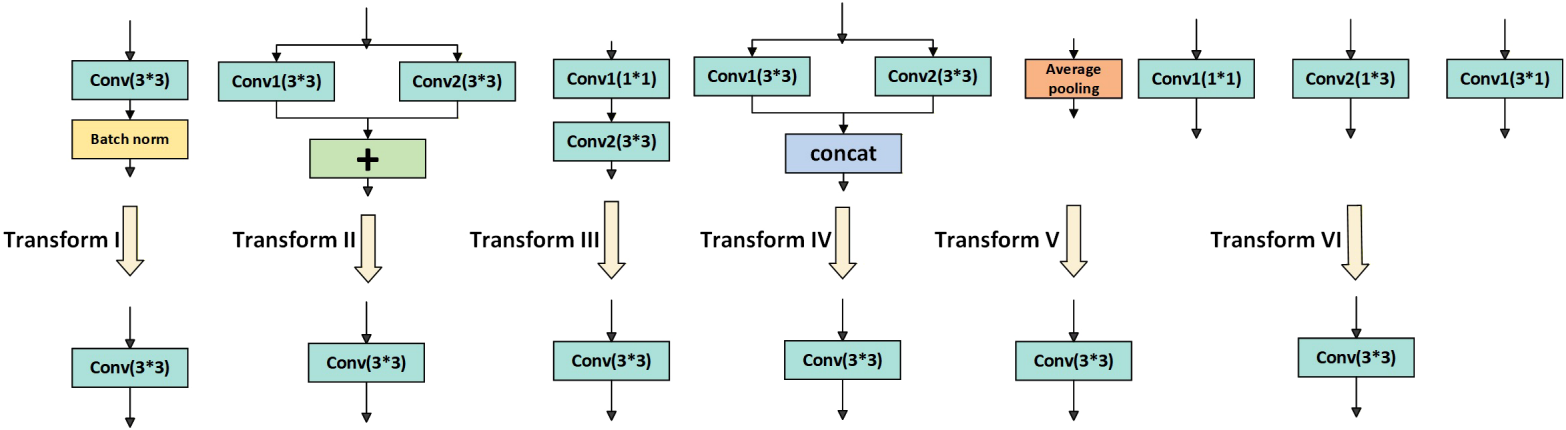
Six equivalent transformations from multi-branch training to single-convolution inference.

While the multi-branch structure provides higher accuracy for lychee canopy recognition, it leads to a surge in computation during inference, running counter to the efficient deployment requirements of UAV vision models. To address this drawback, the MFE block decouples multi-branch enhanced training from efficient single-convolution inference by applying six equivalent-transformation rules (**Figure 6**). Exploiting the linearity properties of convolution, the complex branch structure is equivalently converted into a single convolutional layer, thereby guaranteeing inference efficiency.

Transformation I is Conv–BN fusion, i.e., the fusion of a convolutional layer and its subsequent batch-normalization layer. By mathematical derivation, the BN normalization parameters are absorbed into the convolutional kernel and bias, thus avoiding extra computation at inference. For each output channel *j*, the mathematical expressions are given in Equations 6, 7.

(6) $$F_{j}^{\prime }{,}_{:,:,:}\leftarrow \frac{\gamma_{j}}{\sigma_{j}}F_{j,:,:,:}$$

(7) $$b_{j}^{\prime }\leftarrow \beta_{j}-j\cdot \frac{\gamma_{j}}{\sigma_{j}}\mu_{j}$$

Here, $F\in {\mathbb{R}}^{D\times C\times K\times K}$ denotes the convolution kernel, $b\in {\mathbb{R}}^{D}$ denotes the bias, *µ_j_*and *σ_j_*denote the mean and standard deviation in batch normalization, *γ_j_*and *β_j_*denote the learnable scale and shift (gamma and beta) parameters.

Transformation II is branch-addition merging, i.e., summing the outputs of multiple convolutional branches with identical configurations and merging them into a single convolution. If two (or more) convolutional layers with the same configuration produce outputs that are added, they can be merged into one equivalent convolution, as shown in Equation 8.

(8) $$F^{\prime }\leftarrow F^{\left(1\right)}+F^{\left(2\right)};\;b^{\prime }\leftarrow b^{\left(1\right)}+b^{\left(2\right)}$$

Among, $F^{\left(1\right)},F^{\left(2\right)}\in {\mathbb{R}}^{D\times C\times K\times K}$ denotes the convolution kernel, $b^{\left(1\right)},b^{\left(2\right)}\in {\mathbb{R}}^{D}$ denotes the bias.

Transformation III is the 1×1–K×K sequential merging, which equivalently converts the serial structure of a 1×1 conv–BN followed by a K×K conv–BN into a single K×K convolution by reorganizing kernel dimensions to reduce computational steps. Let the 1×1 convolution kernel be *F*^(1)^ and the K×K convolution kernel be *F*^(2)^, the merged convolution kernel is Equation 9:

(9) $$F^{\prime }=F^{\left(2\right)}*\text{TRANS}(F^{\left(1\right)})$$

the merged bias is Equation 10:

(10) $$b^{\prime }=b^{\left(2\right)}+\text{SUM}(F^{\left(1\right)})\cdot b^{\left(1\right)}$$

Here, TRANS (*F*^(1)^) denotes the transpose of *F*^(1)^, and SUM (*F*^(1)^) denotes the sum of all elements of *F*^(1)^.

Transformation IV is depthwise-concatenation merging: branches concatenated along the outputchannel dimension are merged into a single convolutional layer. In the MFE block, this merging is used to handle branches of different structures (for example, 1×1 convolutions and K×K convolutions). After concatenating their outputs along the channel dimension, they can be represented as one large convolution. Suppose there are *N* convolutional branches and the *i*-th branch has kernel $F^{\left(i\right)}\in {\mathbb{R}}^{D_{i}\times C\times K\times K}$ and bias $b^{\left(i\right)}\in {\mathbb{R}}^{D_{i}}$,the merged convolution kernel and bias are given by expressions Equations 11, 12.

(11) $$F^{\prime }=\text{CONCAT}(F^{\left(1\right)},F^{\left(2\right)},\ldots ,F^{\left(N\right)})\in {\mathbb{R}}^{(\sum_{i=1}^{N}D_{i})\times C\times K\times K}$$

(12) $$b^{\prime }=\text{CONCAT}(b^{\left(1\right)},b^{\left(2\right)},\ldots ,b^{\left(N\right)})\in {\mathbb{R}}^{\sum_{i=1}^{N}D_{i}}$$

Transformation V converts average pooling into an equivalent convolution, thereby eliminating pooling computation overhead at inference. For an input feature map $X\in {\mathbb{R}}^{C\times H\times W}$, its average pooling output is given by Equation 13.

(13) $$Y_{c}=\frac{1}{H\cdot W}\sum_{i=1}^{H}\sum_{j=1}^{W}X_{c,i,j}$$

When converting this operation into a convolution, we construct a convolution kernel $F\in {\mathbb{R}}^{C\times C\times K\times K}$ that satisfies Equation 14,

(14) $$F_{c,c^{\prime },i,j}=\{\begin{array}{ll} \frac{1}{H\cdot W}, & \text{if }c=c^{\prime }\text{ and }i=\frac{K}{2},j=\frac{K}{2}\\ 0, & \text{otherwise} \end{array}$$

Among them, *K* denotes the convolution kernel size. Physically, this convolution kernel implements the operation that the response of each output channel *c* equals the global average value of the corresponding input channel *c*.

Transformation VI is the multi-scale convolution equivalence, which maps convolutions of different kernel sizes to a unified K×K convolution via zero-padding, enabling multi-scale kernels to be fused into a single equivalent K×K kernel and thus unifying multi-scale feature fusion with inference efficiency. Given an input convolution kernel $F\in {\mathbb{R}}^{D\times C\times k_{h}\times k_{w}}$ and a target kernel size *K* × *K*, it is expanded to *F*^′^ ∈ R*^D^*×*^C^*×*^K^*×*^K^* by zero-padding as defined in Equation 15.

(15) $${F^{\prime }}_{d,c,i,j}=\{\begin{array}{ll} F_{d,c,i-H_{p},j-W_{p}} & \text{if }(i-H_{p},j-W_{p})\in [0,k_{h}-1]\times [0,k_{w}-1]\\ 0 & \text{otherwise} \end{array}$$

Where $H_{p}=\frac{K-k_{h}}{2}$, $W_{p}=\frac{K-k_{w}}{2}$ denotes the number of padded pixels on the top, bottom, left and right, d∈[0,D−1], c∈[0,C−1], i,j∈[0,K−1].

The MFE block innovatively adopts four functionally complementary parallel branches together with six equivalence-transform rules. By employing a K×K main branch, a 1×1 branch, an average-pooling branch and a 1×1–K×K sequential branch, the block comprehensively captures multiscale textures and structural features of lychee canopies, addressing the limited expressiveness of conventional single-kernel convolutions. Simultaneously, through the equivalence transforms the complex multi-branch training-time structure can be losslessly converted into a single convolution for inference, substantially reducing inference overhead while preserving accuracy. This design balances precision and efficiency and is well suited for real-time deployment on UAV edge devices.

### 3.4 SD module

Downsampling is a critical step for enlarging the receptive field, but it is also the primary stage where information about small objects is lost. In lychee canopy segmentation, the boundary details and sparse branches occupy very few pixels. Conventional models, such as YOLOv5 and YOLOv8, typically use a 3×3 standard convolution to perform spatial downsampling and channel expansion simultaneously. This “strongly coupled” design requires a single convolutional layer to handle two operations of different natures, forcing the model to discard spatial details before channel features are fully integrated,often causing the loss of high-frequency details such as canopy edges and small gaps. In the complex scenario of lychee orchards, where fruit tree density varies and branches are intertwined, this operation causes high-frequency details to be excessively smoothed, leading to blurred canopy edges and missed detections of small gaps. Furthermore, the computational redundancy of these standard convolutions hinders the inference speed required for UAVs.

**Figure 7:**
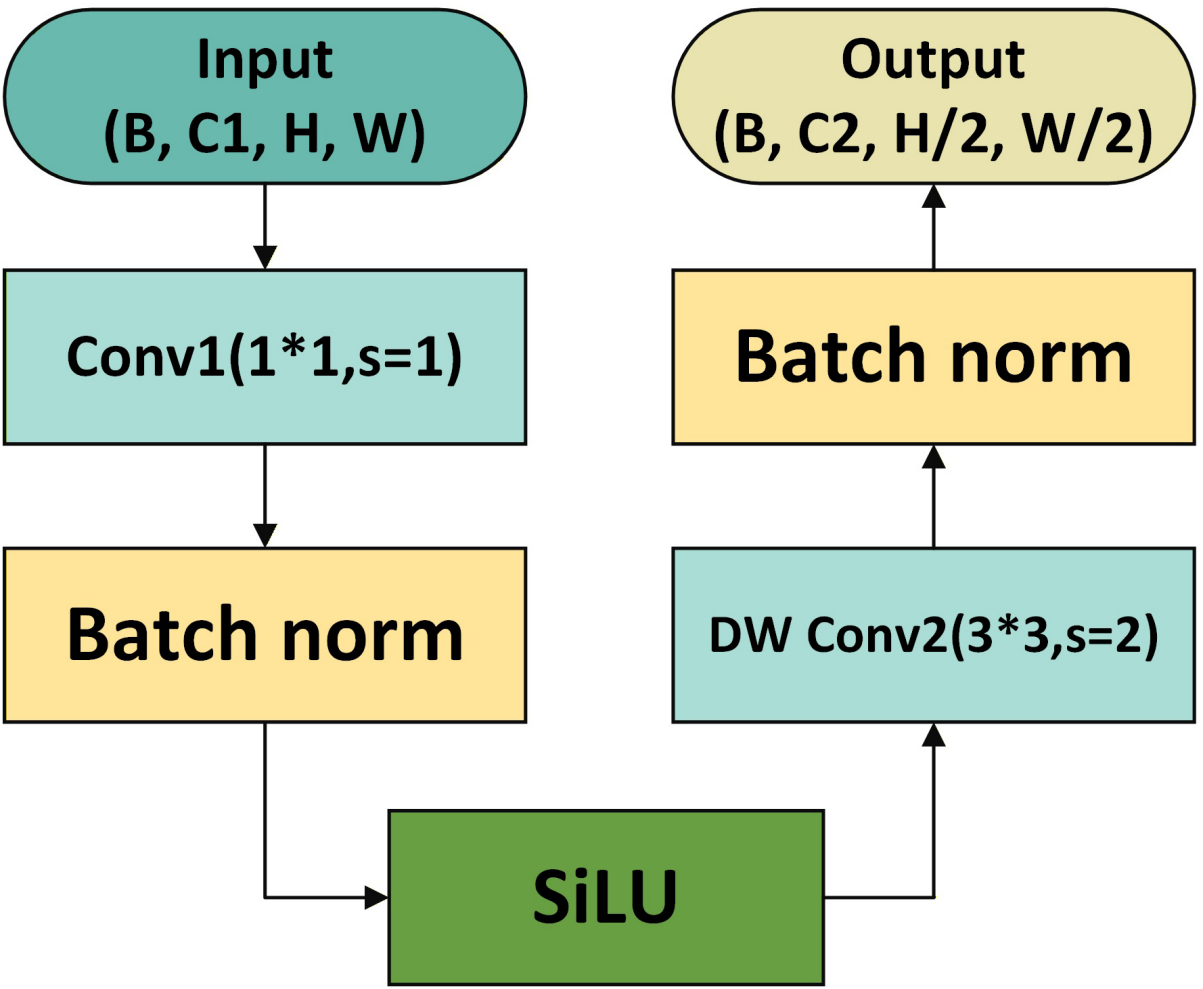
The core process of feature preprocessing and downsampling for the SD module. For an input of size (B,C1,H,W), it first undergoes a 1×1 convolution with stride 1 and batch normalization to adjust the channel dimensions; after SiLU activation, it achieves downsampling via a 3×3 depthwise separable convolution with stride 2 and batch normalization, ultimately outputting a feature map of size (B,C2,H/2,W/2).

To solve this problem, we propose the Spatial-Channel Decoupled (SD) module (**Figure 7**). The core design philosophy is “preserve first, compress later”. Unlike the traditional approach, the SD module adopts a decoupled strategy: it first performs channel mixing (via pointwise convolution) to retain rich semantic information, and then performs spatial feature extraction and compression (via depthwise convolution). This reverse order effectively reduces feature loss and computational redundancy, making it highly suitable for the real-time constraints of UAV-based monitoring.

Let the input feature map be $X\in {\mathbb{R}}^{C_{\text{in}}\times H\times W}$ (where *C*_in_ denotes the number of input channels and (*H, W*) denotes the input spatial size). The SD downsampling process can be represented in two steps:

The first step is channel-dimension adaptation, which uses a 1×1 convolution to perform channel interaction and dimensional adjustment independently, optimizing inter-channel information flow and avoiding channel interference during spatial compression. This step can be expressed as Equation 16:

(16) $$X_{1}=\sigma (\text{BN}(W_{1}*X+b_{1}))$$

where $W_{1}\in {\mathbb{R}}^{C_{\text{out}}\times C_{\text{in}}\times 1\times 1}$ is the 1×1 convolution kernel, BN (·) denotes batch normalization, *σ* denotes the SiLU activation function, and *C*_out_ is the target number of output channels. This step only adjusts the channel dimension and does not change the spatial resolution, ensuring thorough channel-wise feature interaction.

The second step is spatial downsampling, which adopts a depthwise convolution whose number of groups equals the number of output channels to perform spatial scale reduction independently and to reduce interference between channel and spatial operations. This step can be expressed as Equation 17:

(17) $$X_{\text{out}}=\text{BN}(W_{2}⊛X_{1}+b_{2})$$

where $W_{2}\in {\mathbb{R}}^{C_{\text{out}}\times 1\times K\times K}$ is the depthwise convolutional kernel, *K* denotes the kernel size, ⊛ specifically denotes the depthwise discrete convolution operator to distinguish it from standard convolution, and stride *s* ≥ 1 implements the downsampling. This step focuses only on spatial compression and does not change the channel dimension, maximizing preservation of the channel features and spatial details optimized in the first step.

The two steps operate cooperatively to form the complete downsampling process, ultimately outputting $X_{\text{out}}\in {\mathbb{R}}^{C_{\text{out}}\times H_{\text{out}}\times W_{\text{out}}}$. The core idea is the decoupling logic of first optimizing channels and then compressing space, which avoids feature loss caused by mutual interference between channel transformation and spatial compression in traditional single-step downsampling. The SD module breaks through the dual bottlenecks of computational redundancy and feature loss caused by the coupling of channel transformation and spatial compression in conventional downsampling, providing an efficient feature-scale compression scheme for real-time object detection. The edge information of the lychee canopy is fully preserved, ensuring the excellent segmentation effect of the UAV real-time monitoring mission.

## 4 Results

In the performance evaluation of MFD-YOLO, the input image size was uniformly set to 640×640 pixels. The model was trained and compared using multiple combinations of hyperparameters, with mAP50 selected as the primary evaluation metric. A total of 200 training epochs and a batch size of 8 were finally adopted to ensure optimal convergence, and an early stopping mechanism was introduced to suppress overfitting. To ensure optimization stability during training, SGD was selected as the optimizer, with an initial learning rate of 0.01. A cosine annealing learning rate schedule was applied to balance convergence speed and training stability. No data augmentation strategies were employed in the experiments. The model weights were randomly initialized, and the random seed was set to 42. Furthermore, each model was independently tested n=3 times to evaluate the stability of the results. For the segmentation task, the mask downsampling ratio was set to 4 to balance memory consumption and segmentation accuracy.

### 4.1 Comparison with mainstream models

As a benchmark in object detection, the YOLO series has undergone extensive evolution. YOLOv5 achieves a balance between accuracy and speed through the CSP backbone and PANet feature fusion, and has been widely used for orchard crop detection. YOLOv8 further optimizes the end-to-end training pipeline for instance segmentation and achieves high average precision. These “single-stage detection” architectures fundamentally address the issue of slow inference in earlier models, enabling real-time deployment in agricultural applications.

Compared with other YOLO models, MFD-YOLO demonstrates superior adaptability to agricultural segmentation tasks, owing to its stronger multi-scale feature extraction ability and its dynamic label assignment strategy in the detection head, making it particularly suitable for scenarios with mixed-scale lychee tree canopies. **Table 1** presents comparisons with mainstream YOLO models. In the lychee canopy segmentation task, MFD-YOLO exhibits notable performance improvements over existing segmentation models. Compared with YOLOv8-seg-p6, mAP50 increases by 3.1 percentage points; compared with YOLOv9-c-dseg, mAP50 increases by 2.3 percentage points and precision improves by 3.6 percentage points. These improvements are achieved without significant increases in parameter count or GFLOPs, while achieving lower latency and simultaneous improvements in both precision and recall. It is important to clarify that the latency benchmarks reported in **Table 1** were conducted on a high-performance server; therefore, the reported latency of 15.5 ms represents ‘server-side real-time’ performance. Although the model demonstrates a significant reduction in theoretical computational redundancy and algorithmic latency, future validation on representative edge hardware remains necessary.

**Table 1:** Comparison with mainstream models.

Model	Param	FLOPs	Latency	Precision	Recall	mAP50
	(/M)	(/G)	(/ms)	(/%)	(/%)	(/%)
YOLOv3 (Redmon and Farhadi, 2018)	103.69	282.7	8.6	57.50±1.00	61.35±1.65	59.75±0.95
YOLOv3-spp (Redmon and Farhadi, 2018)	104.74	283.6	5.7	57.45±0.85	62.15±0.75	60.90±1.30
YOLOv3-tiny (Redmon and Farhadi, 2018)	12.13	18.9	2.1	60.20±1.10	59.90±1.80	60.15±1.55
YOLOv5	2.51	7.1	7.9	63.45±1.55	60.90±1.00	64.70±0.20
YOLOv5-p6	4.13	7.2	11.3	64.50±1.70	61.10±0.70	65.85±1.15
YOLOv6 (Li et al., 2022)	4.23	11.8	8.1	63.45±0.25	61.40±2.10	64.60±1.30
YOLOv8-seg (Wang et al., 2024)	3.26	12.1	8.6	62.65±2.05	62.35±2.15	65.00±0.70
YOLOv8-seg-p6	5.10	12.0	13.1	65.30±1.40	61.85±1.45	65.80±0.40
YOLOv9-c-dseg (Wang et al., 2025)	57.47	368.6	17.3	65.00±0.80	63.05±0.45	67.35±0.35
MFD-YOLO	59.31	389.0	15.5	68.85±0.25	62.85±0.85	69.40±0.10

YOLOv5 has been developed by Ultralytics but has not yet been formally published in academic papers. The device used for the above benchmark test has an Intel(R) Xeon(R) Gold 6240R CPU @ 2.40GHz with 100 GB of RAM, and an NVIDIA Tesla A800 GPU with 80 GB of VRAM. Results are reported as Mean ± Error based on multiple independent runs.

**Figure 8:**
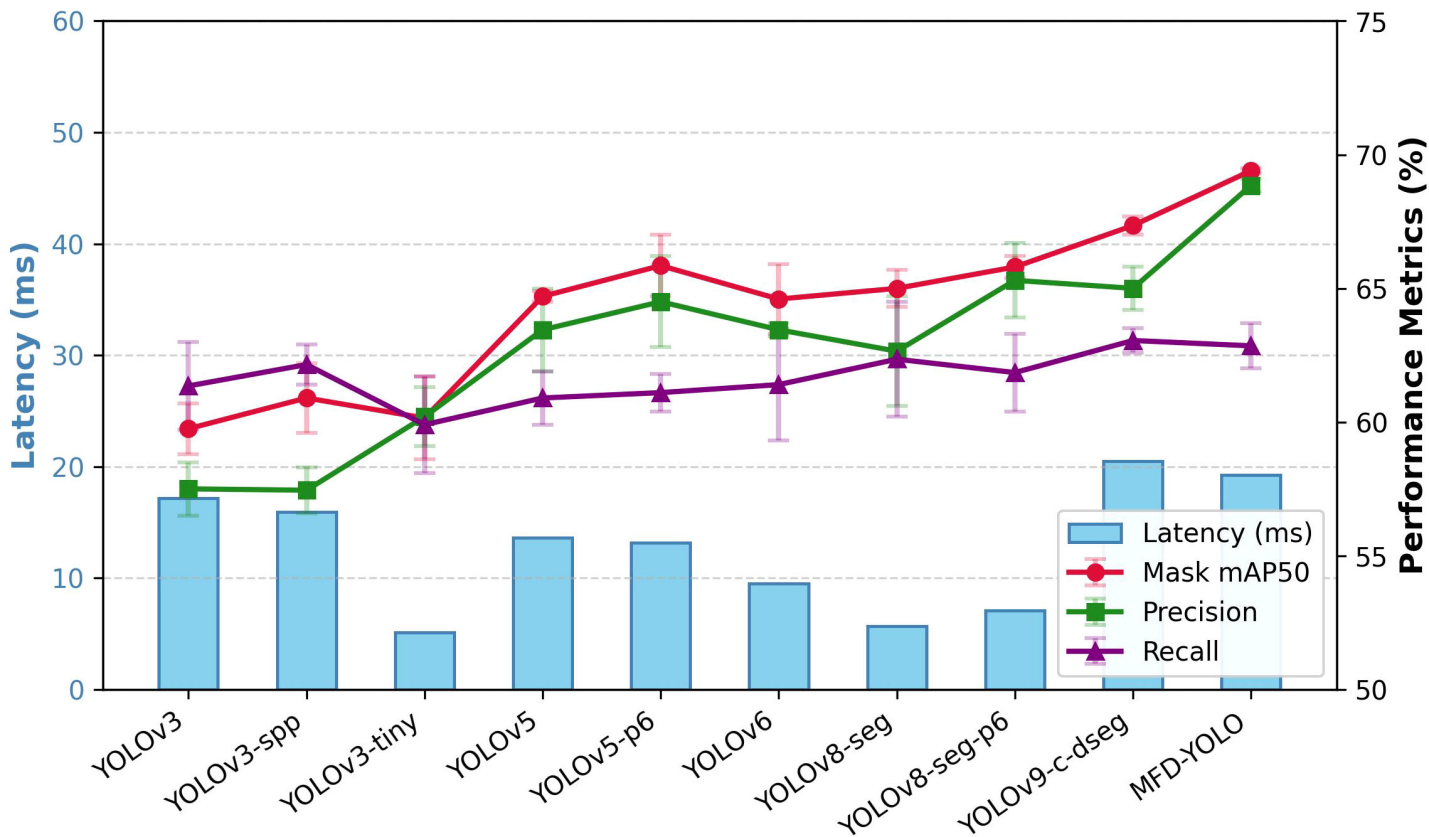
The line-bar chart of the model’s precision, recall, mAP50, and latency intuitively reflects the improvement in model performance. The device used for the above benchmark test has an Intel(R) Xeon(R) Gold 6240R CPU @ 2.40GHz with 100 GB of RAM, and an NVIDIA Tesla A800 GPU graphics card with a VRAM of 80GB.

This breakthrough benefits from the synergy between the multi-branch re-parameterization design of the MFE block and the spatial-channel decoupled downsampling strategy of the SD module. The MFE block enhances feature diversity through its four-branch parallel structure, enabling the model to better capture fine-grained branches and occlusions in complex orchard environments, directly contributing to the mAP50 and precision gains. The SD module effectively preserves canopy edge details while reducing computational redundancy, maintaining a high recall of 63.6. Its latency performance surpasses most YOLO models of similar accuracy, and even when compared with lighter YOLO variants, the increase in latency remains minimal. These results collectively validate the engineering value of the proposed “feature enhancement + efficient downsampling” design for UAV edge deployment in agriculture. **Figure 8** shows the comparison of precision, recall, mAP50 and latency among different models, where MFD-YOLO demonstrates superior accuracy-efficiency trade-off.

### 4.2 Ablation experiment

The primary contribution of this experiment lies in introducing the MFE and SD modules, which respectively optimize feature extraction and downsampling to achieve a balance between accuracy and efficiency. To quantify their individual contributions and synergistic effects, ablation studies were conducted using YOLOv9-c-dseg as the baseline. The results are summarized in **Table 2** 2.When only the MFE block is introduced, mAP50 increases from 67 to 68.8, while precision and recall increase by 1.3 and 1.7 percentage points, respectively. This demonstrates that the multibranch re-parameterization design effectively enhances canopy feature representation in complex scenes. When only the SD module is used, mAP50 improves to 68.6, and recall increases by 2.2 percentage points, while GFLOPs remain controlled at 388.3. This verifies that the spatial-channel decoupled downsampling strategy successfully reduces redundancy while preserving crucial edge structures and fine branches, yielding significant recall improvement. When both modules are deployed simultaneously, mAP50 further increases to 69.3, and precision improves by 3.6 percentage points, realizing the combined benefits of “MFE-based feature enhancement + SD-based efficient downsampling. “GFLOPs remain around 389, and the latency results further confirm the model” s advantages in efficient real-time deployment. Ultimately, the proposed model achieves a dual breakthrough in accuracy and efficiency for lychee canopy segmentation.

**Table 2:** Ablation experiment.

Strategies	Param (/M)	FLOPs (/G)	Box		Mask	
			Precision (/%)	mAP50 (/%)	Precision (/%)	mAP50 (/%)
YOLOv9-c-dseg	57.47	368.6	63.85 ± 0.45	67.30 ± 0.40	65.00 ± 0.80	67.35 ± 0.35
MFE only	65.59	395.2	65.35 ± 0.35	68.70 ± 0.70	66.75 ± 0.25	68.90 ± 0.60
SD only	59.29	388.3	64.65 ± 0.85	68.10 ± 0.60	66.20 ± 0.70	68.60 ± 0.20
MFD-YOLO	59.31	389.0	65.85 ± 0.45	68.80 ± 0.20	68.85 ± 0.25	69.40 ± 0.10

The device used for the above benchmark test has an Intel(R) Xeon(R) Gold 6240R CPU @ 2.40GHz with 100 GB of RAM, and an NVIDIA Tesla A800 GPU with 80 GB of VRAM. Results are reported as Mean ± Error based on multiple independent runs.

We observe that the synergistic collaboration between the MFE block and the SD module enables a direct improvement in the mAP50 metric, while the inference efficiency remains at the original level, ultimately achieving a balance between accuracy and efficiency.

### 4.3 Segmentation performance

The MFD-YOLO model, by virtue of the collaborative optimization of the dual MFE block and SD module, demonstrates exceptional scene adaptability and detailed segmentation accuracy in lychee canopy segmentation tasks. The dataset adopted in this experiment is systematically constructed, comprehensively covering lychee orchard scenarios under various conditions: it includes not only high-density lychee orchards with small plant spacing and complex orchards shielded by greenhouse frames and plastic films, but also multi-scale planting scenarios in hilly areas characterized by significant terrain undulations and large variations in fruit tree planting density. These scenarios not only require the model to accurately segment the densely intertwined canopy boundaries, but also to cope with multiple interferences such as occlusion shadows, perspective distortion caused by terrain differences, and variations in foliage density, posing significant challenges to the pixel-level segmentation of lychee canopies. Nevertheless, the MFD-YOLO model can still stably output high-precision segmentation results, fully verifying its robustness in complex agricultural scenarios.

**Figure 9:**
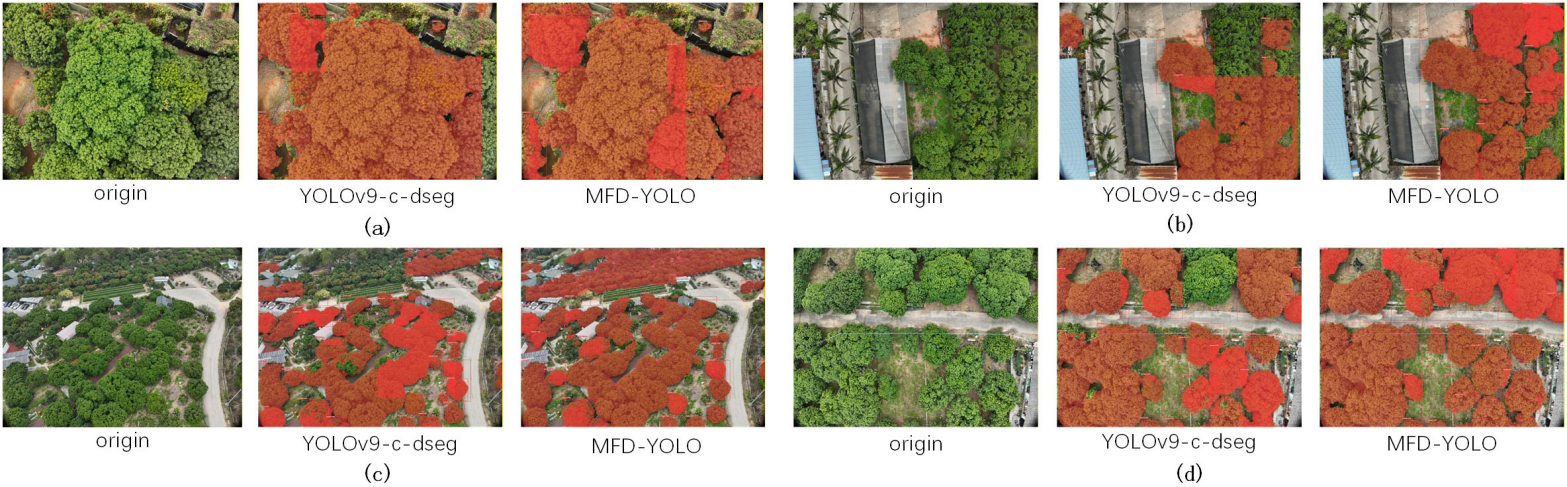
Segmentation Results of Lychee Orchards with YOLOv9-c-dseg and MFD-YOLO Under Different Scenarios. **(a)** High-density lychee orchards; **(b)** Lychee orchards shaded by greenhouses; **(c)** Lychee orchards with great topographical variations; **(d)** Lychee orchards with significant density variations.

As can be observed from the segmentation maps (**Figure 9**), the model achieves accurate and complete canopy segmentation across various scenarios. The segmentation boundaries are highly consistent with the actual canopy contours, with almost no missed detections or false detections. This excellent performance is attributed to the multi-dimensional feature enhancement of the MFE block and the efficient preservation of edge information by the SD module during spatial downsampling. Through their efficient collaboration, the two modules enable the model to adapt to all scene variations—from dense clusters to isolated individuals and from occluded to unoccluded scenarios—providing high-precision and highly reliable visual technical support for the intelligent monitoring and management of lychee orchards.

### 4.4 Heatmap analysis based on EigenCAM

To visually elucidate the effective attention of MFD-YOLO to both local and global features, this study employs the EigenCAM Muhammad and Yeasin (2020) (Eigen Class Activation Map) technique to conduct a comparative visualization analysis of the pre-trained weights of MFD-YOLO and the baseline YOLOv9-c-dseg. Unlike traditional gradient-dependent visualization methods, EigenCAM computes the principal components of feature maps to identify regions with high activation values within specific layers. This process reveals the spatial structures emphasized by the network, thereby providing an unbiased perspective of the learned spatial representations. Given that accurate segmentation of canopy edges requires the capture of intricate textural information and fine boundary details, the heatmap analysis is specifically focused on the P3 layer, which retains the highest spatial resolution.

**Figure 10:**
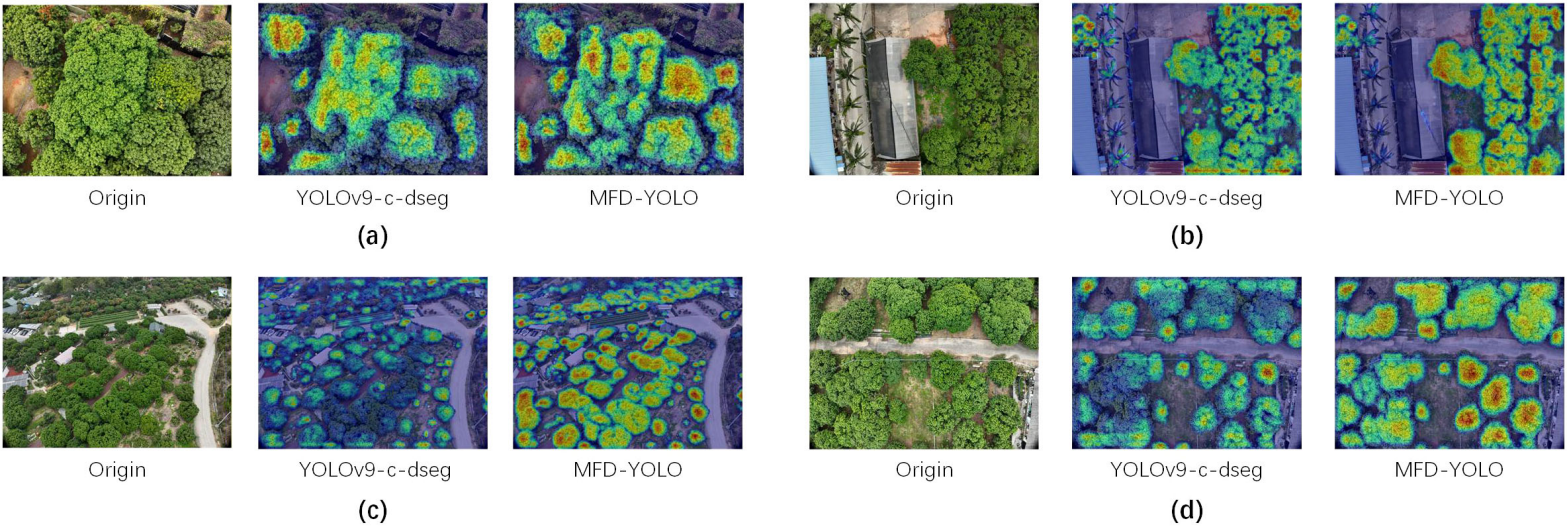
Comparison of YOLOv9-c-dseg and MFD-YOLO heatmaps based on EigenCAM across four complex scenarios: **(a)** High-density lychee orchards, **(b)** Lychee orchards shaded by greenhouses, **(c)** Lychee orchards with great topographical variations, and **(d)** Lychee orchards with significant density variations. Compared to the baseline, MFD-YOLO demonstrates more precise activation coverage along the canopy edges and finer response to local leaf textures.

The visualization results are presented in **Figure 10**. A comparative analysis with the baseline model reveals that MFD-YOLO demonstrates superior performance in edge delineation and local texture recognition. Notably, the regions of high activation align precisely with the edges of the lychee canopies, showing a high degree of congruence with local textural patterns. This precise attention distribution is attributed to the synergistic effect of the MFE block and the SD module. Specifically, the MFE block enhances the capture of fine-grained leaf information during the feature extraction phase, while the SD module effectively prevents the loss of minute targets and edge details during the subsequent downsampling process. The results indicate that MFD-YOLO is capable of achieving precise target acquisition and segmentation across canopy scenarios of varying scales. Furthermore, it provides adequate attention even at shallower network levels, ensuring effective monitoring of multi-scale targets for UAV-based orchard applications.

## 5 Discussion

In recent years, relevant studies have attempted to apply real-time algorithms, such as YOLACT Chen et al (2018), to lychee canopy segmentation tasks. By generating masks via a linear combination of prototypes, these methods achieve high inference speeds and reasonable accuracy. However, the resulting masks often exhibit boundary leakage or over-smoothing, resulting in suboptimal performance regarding the fine segmentation details of lychee canopy edges. Some studies also focus on the lightweighting and efficiency of models. For instance, the LGVMYOLOv8n Han et al. (2025) model proposed by Han et al. employs lightweight operators such as GSConv (Ghost-Shuffle Convolution) to significantly reduce parameters and FLOPs, substantially raising efficiency. While effective in apple instance segmentation, in high-density occlusion scenarios typical of lychee canopies, such models inevitably sacrifice a degree of feature representational capability to maintain lightweight performance, leading to the loss of edge information.

To address key challenges in lychee canopy segmentation—specifically poor adaptability to complex environments, imprecise segmentation boundaries, and low inference efficiency—we propose the MFD-YOLO model. Distinct from prior research, our model adopts a decoupled head architecture and structural re-parameterization techniques (MFE block). Furthermore, by utilizing the SD module to explicitly preserve spatial details during downsampling, the model improves canopy edge fidelity with very little computational cost. This achieves a superior balance between efficient deployment and the retention of fine-grained features, ensuring precise segmentation of lychee canopy edges during UAV missions.

MFD-YOLO exhibits remarkable performance in lychee canopy recognition, achieving significant breakthroughs in both precision and efficiency by prioritizing fine-grained feature capture and optimizing the trade-off between efficient design and feature preservation. This optimized model ensures robust edge segmentation and scene adaptability, thereby directly supporting intelligent orchard management tasks such as tree counting, growth monitoring, and yield estimation. Furthermore, its efficient nature meets the engineering requirements for real-time UAV monitoring, significantly lowering the technical deployment costs of smart agriculture. Despite the promising performance of MFD-YOLO in balancing inference efficiency and segmentation accuracy, it is necessary to consider and clarify the limitations of this study for future improvements. First, the current model is trained on a relatively small dataset comprising UAV-acquired images captured under specific geographical and weather conditions; this limited distribution may restrict the model’s robustness under extreme conditions. Second, in extreme cases where canopy boundaries are completely occluded, the model may still exhibit under-segmentation. Resolving this ambiguity may require multi-modal approaches, such as incorporating 3D LiDAR Omasa et al. (2007) point clouds to provide depth geometry. Finally, it must be acknowledged that there is a certain gap between the current hardware evaluation and actual edge deployment. The evaluation of the model’s metrics in this study was conducted on a high-performance server, whereas real-world UAVs are constrained by various conditions. Therefore, the actual onboard inference latency may deviate from our theoretical benchmarks. Future research directions will focus on cross-species transfer and practical UAV deployment.

The study utilized a dedicated dataset of high-resolution (5280×3956) UAV-acquired RGB images covering multiple growth stages and planting densities. While the current validation is focused on lychee orchards, whose morphological features are representative of subtropical evergreen fruit trees, future work aims to explore the model’s transferability to other species such as citrus and mango. To achieve this, we plan to expand the dataset’s diversity and apply few-shot transfer learning strategies by selecting representative annotated samples and employing a layered training approach —freezing the initial general feature layers while fine-tuning the head with customized regularization techniques—to adapt to specific species characteristics without overfitting. Concurrently, although the efficient principles of MFD-YOLO have been experimentally verified on high-performance servers, future research will focus on migrating the model to embedded edge devices to rigorously evaluate its inference efficiency under real-world flight conditions.

## 6 Conclusions

This study addresses the practical needs of precision management in lychee orchards and explores the broad potential of UAV technology in precision agriculture. Traditional segmentation models provide feasible solutions for UAV edge deployment, but their limited feature expression, difficulty adapting to complex orchard environments, and insufficient inference efficiency prevent them from balancing accuracy and efficient inference requirements.

To support downstream tasks such as precision spraying, pest detection, and yield prediction, this study focuses on lychee canopy segmentation and proposes a efficient model—MFD-YOLO. The model innovatively integrates the MFE multi-branch re-parameterization block and the SD spatial-channel decoupled downsampling module. The MFE block captures multi-scale features through a four-branch parallel structure and is re-parameterized into a single convolution for inference without additional computational cost. The SD module reduces feature loss and computational redundancy through its decoupled channel-first downsampling strategy. Evaluation results show that the model reaches an mAP50 of 69.3, outperforming YOLOv8-seg-p6 and YOLOv9-c-dseg by at least 2.3 percentage points. It achieves accurate segmentation in complex orchard environments while maintaining a parameter size and latency suitable for UAV edge deployment.

The proposed MFD-YOLO model already supports intelligent orchard management tasks such as canopy-based tree counting and precision spraying, substantially improving UAV perception accuracy. Future work will explore cross-species adaptation by expanding multi-species datasets and incorporating few-shot transfer learning strategies to enable generalization to more economic crops. Furthermore, the “feature enhancement + efficiency optimization” design paradigm introduced in this study offers a reusable framework for developing edge-deployable segmentation models for UAVs, with potential to accelerate large-scale applications of vision-based smart agriculture.

## Data Availability

The original contributions presented in the study are included in the article/supplementary material. Further inquiries can be directed to the corresponding author.
